# Impact of explicit fluoride-related language in prefectural dental health ordinance on fluoride mouth-rinse programs dissemination in Japan: a quasi-experimental study

**DOI:** 10.3389/froh.2026.1780911

**Published:** 2026-03-02

**Authors:** Manami Hoshi-Harada, Chieko Taguchi, Yoichi Ishizuka, Azusa Ishiguro, Yusuke Matsuyama, Jun Aida, Ken Osaka, Kenji Takeuchi

**Affiliations:** 1 Department of International and Community Oral Health, Tohoku University Graduate School of Dentistry, Miyagi, Japan; 2 Department of Community Oral Health, Nihon University School of Dentistry at Matsudo, Chiba, Japan; 3 Department of Epidemiology and Public Health, Tokyo Dental College, Tokyo, Japan; 4 Department of Dental Hygiene, Tsurumi Junior College, Kanagawa, Japan; 5 Department of Dental Public Health, Graduate School of Medical and Dental Sciences, Institute of Science Tokyo, Tokyo, Japan; 6 Division of Statistics and Data Science, Liaison Center for Innovative Dentistry, Tohoku University Graduate School of Dentistry, Miyagi, Japan

**Keywords:** dental caries, fluorides, health inequities, health policy, implementation science, program evaluation, public health, school health services

## Abstract

**Objectives:**

To estimate the impact of explicit fluoride-related language in prefectural dental health ordinance on the dissemination of school-based fluoride mouth-rinse (S-FMR) programs.

**Methods:**

A longitudinal ecological design was employed to analyze prefectural-level panel data from Japan spanning 2007 to 2018. Ordinances enacted between 2010 and 2014 were categorized by the presence of explicit fluoride-related terms: 1) explicit mention of “fluoride mouth-rinse” (FMR group); 2) explicit mention of “fluoride application” (FA group); and 3) no fluoride-related policy language (NF group). The outcome was the proportion of children aged 4–15 years participating in S-FMR programs. Total prefectural income per year, prefectural mean age, and prefectural mean number of decayed, missing, or filled primary teeth (dmft) among 3-year-old children were included as covariates. The Callaway and Sant'Anna Difference-in-Differences (CSDID) method was applied to estimate the average treatment effects on the treated (ATT) for the FMR and FA groups under a conditional parallel trends assumption.

**Results:**

A total of 39 prefectures were analyzed. The pre–post increase in S-FMR participation was greater in the FMR group than in the FA or NF groups, with comparable differences (FMR: 12%; FA and NF: 5% each). In the CSDID analysis, both the FMR and FA groups showed a significant increase in S-FMR participation compared with the NF group, with a larger effect in the FMR group [FMR: 8% (95% CI: 2%–15%); FA: 5% (95% CI: 0%–9%)]. The event-study estimates indicated that the effects strengthened over time, particularly in the FMR group.

**Conclusions:**

Prefectural dental health ordinances explicitly refer to fluoride, particularly FMR, are associated with a greater dissemination of the proportion of children participating in S-FMR programs. These findings suggest that more specific and explicit policy language in dental health ordinances may enhance the dissemination of S-FMR programs.

## 1 Introduction

Untreated dental caries affects more than one-third of the global population and remains the most prevalent noncommunicable disease, constituting a persistent public health concern worldwide (1, 2). In addition, socioeconomic inequality in the incidence of dental caries is evident, underscoring the importance of population-based strategies that do not depend solely on individual effort (3). As a population-based strategy, community water fluoridation is widely recognized as a cost-effective and equitable measure for caries prevention (1). However, many countries, including Japan, have been unable to implement it because of various factors such as cultural resistance and limited political acceptance (1).

A global comparative study indicated that the intake of sugar-sweetened beverages among children and adolescents in Japan is broadly comparable to the global average; moreover, while consumption has increased globally in recent decades, it has shown a declining trend in Japan (4). In addition, Japan's universal health coverage system ensures relatively easy access to dental care (5), and most children and adolescents in Japan brush their teeth at least twice a day, with a prevalence of 87% among those aged 10–14 years (6), which is relatively high internationally (7). Nevertheless, the 12-year-old child's caries experience, as measured by the decayed, missing, and filled teeth (DMFT) index in Japan cannot be regarded as particularly low in international comparison, and the limited use of fluoride-based caries-preventive measures has been cited as one plausible explanation for this discrepancy (5). In Japan, despite the limited implementation of other community-level fluoride applications, school-based fluoride mouth-rinse (S-FMR) programs have been adopted as a population-based preventive strategy (5, 8) to prevent dental caries among children and adolescents (9). Similar programs have been implemented worldwide (10, 11), and since 2023, fluoride mouth-rinse has been included in the World Health Organization (WHO)'s list of essential medicines (10). Furthermore, S-FMR programs are characterized by high cost-effectiveness (12, 13) and their potential to reduce socioeconomic inequalities in dental caries (14, 15).

Although the Japanese government has issued national recommendations for S-FMR implementation (16, 17), program adoption is primarily implemented by municipalities and may be strongly influenced by the policies of their respective prefectures. Previous studies have reported that the implementation of policies at the local government level depends on the extent to which state governments prioritize national goals (18), suggesting that issuing national guidelines alone is unlikely to lead to the dissemination of S-FMR programs in practice. Consistent with this, substantial prefectural variation in S-FMR dissemination has been observed in Japan (8, 14). A previous study in Japan demonstrated an increase in the proportion of children participating in S-FMR programs following the issuance of national guidelines in 2003 (16, 19). However, given the structure of local governance in Japan, this dissemination cannot be attributed solely to national guidelines. Prefectural initiatives, particularly legal or quasi-legal frameworks, such as dental health ordinances, may therefore shape municipal implementation.

Prefectural dental health ordinances, developed after the issuance of national guidelines, have been widely adopted as discretionary regulations in Japan and vary in the specificity of fluoride-related language. However, no longitudinal studies have investigated the impact of prefectural dental health ordinances on the dissemination of S-FMR programs before and after policy implementation. We hypothesized that prefectural dental ordinances that explicitly include fluoride-related languages would promote greater dissemination of S-FMR programs. Using prefecture-level panel data and a quasi-experimental Difference-in-Differences design, we examined the impact of policy language specificity on the proportion of children participating in S-FMR programs.

## 2 Materials and methods

### 2.1 Study design and setting

This study employed a longitudinal ecological design using prefectural-level panel data from Japan spanning 2007 to 2018. We chose 2018 as the endpoint because it was the latest year with available data, and 2007 as the starting year to ensure a period of more than 10 years to assess medium- to long-term trends in S-FMR program dissemination. The unit of analysis was the prefecture because the exposure of interest, such as prefectural dental health ordinances and their policy language, operates at the prefectural level and is expected to influence the dissemination of S-FMR programs through prefecture-to-municipality policy channels. Moreover, for policymakers, decisions regarding ordinance enactment or revision are typically based on aggregated population-level outcomes rather than on individual-level effects (20). The Strengthening the Reporting of Observational Studies in Epidemiology (STROBE) reporting guidelines were followed.

**Figure 1:**
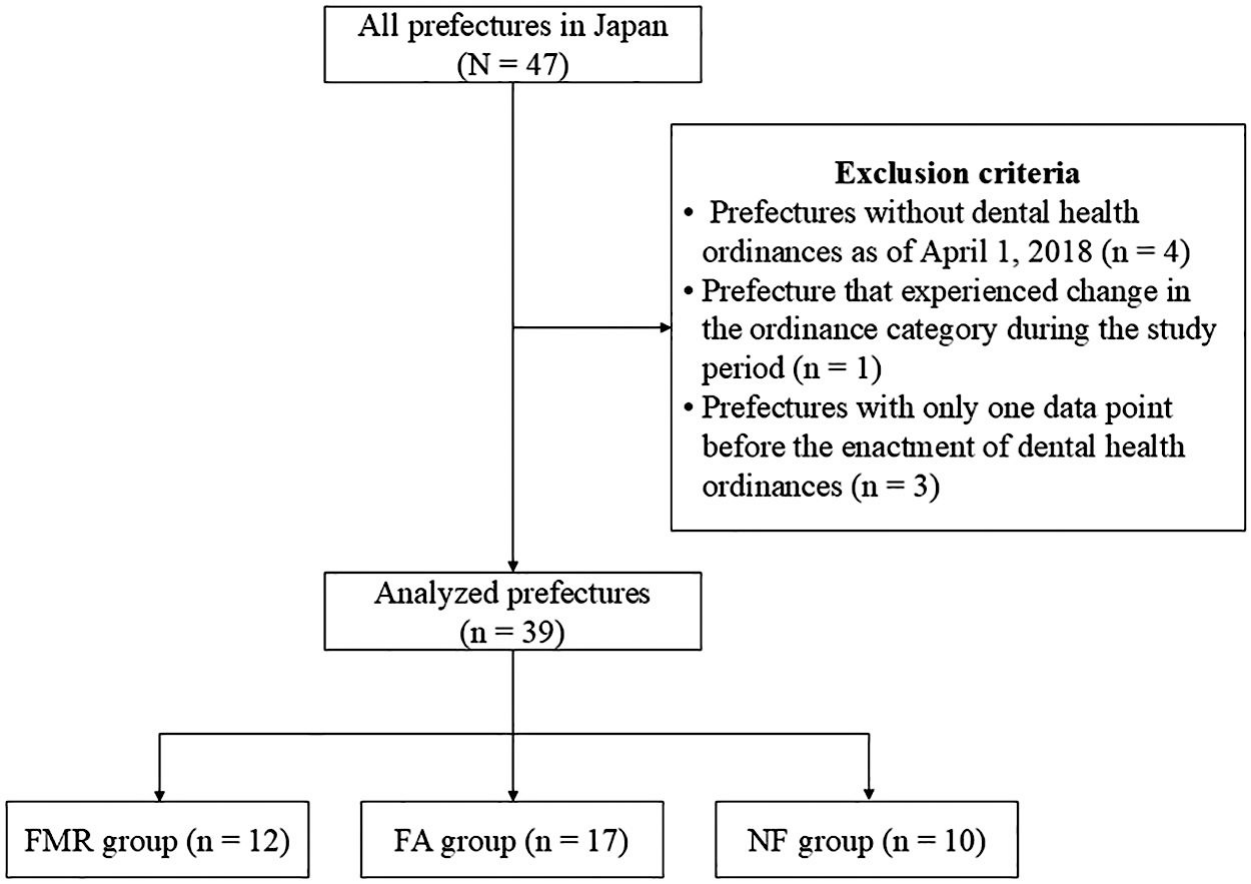
Flowchart of prefectures included in the analysis. FMR group, those explicitly mentioning “fluoride mouth-rinse” in the dental health ordinances; FA group, those explicitly mentioning “fluoride application” in the dental health ordinances; NF group, those without explicit fluoride-related policy language in the dental health ordinances.

The exclusion criteria were as follows: 1) prefectures without dental health ordinances as of April 1, 2018 (*n* = 4), 2) prefecture that experienced change in the ordinance category during the study period (*n* = 1), and 3) prefectures with only one data point before the enactment of dental health ordinances, that is, ordinances enacted in 2008 or 2009 (*n* = 3) (Figure 1). Prefectures without dental health ordinances were excluded because of their small sample sizes and because this study focused on the policy language of such ordinances. Prefectures with only one pre-ordinance data point were excluded because the pre-intervention trends could not be adequately evaluated.

### 2.2 Exposures: policy language in prefectural dental health ordinances

Information on policy language in dental health ordinances from all prefectures in Japan as of April 1, 2018, was obtained from official prefectural websites and the 8020 Promotion Foundation website (21). Prefectural dental health ordinances are discretionary local policy regulations that are not mandated by national law (22). First enacted in 2008, they have since expanded nationwide (22), with 45 of 47 prefectures in Japan having established such ordinances as of March 2023 (21). Although the Act Concerning the Promotion of Dental and Oral Health in Japan was promulgated in 2011 (23, 24), the widespread enactment of these ordinances preceded national legislation, reportedly accelerating its establishment (22). These prefectural ordinances play an important role in reducing regional inequalities in oral health and ensuring coordination with other policies (22). In this study, prefectural dental health ordinances enacted between 2010 and 2014 were categorized into three groups based on the presence of explicit fluoride-related policy language: 1) explicit mention of “fluoride mouth-rinse” (FMR group); 2) explicit mention of “fluoride application” (FA group); and 3) no explicit fluoride-related policy language (NF group).

### 2.3 Outcome: dissemination of S-FMR programs

The outcome, defined as the dissemination of S-FMR programs, was the annual proportion of children aged 4–15 years participating in S-FMR programs in each prefecture, calculated by dividing the number of children participating in S-FMR programs by the total number of eligible children aged 4–15 years.

Data on the number of children participating in S-FMR programs were collected on a biennial basis. Data for 2007, 2009, and 2011 were derived from joint surveys conducted by the Nonprofit Japanese Conference on the Promotion of the Use of Fluoride in Caries Prevention, the 8020 Promotion Foundation, and the WHO Collaborating Centre for Translation of Oral Health Science (8, 25, 26). Data for 2013 and 2015 additionally included collaborations with the Japan Association of School Dentists (27, 28). The 2018 data originated from the Ministry of Health, Labour, and Welfare (MHLW) (29). The total number of eligible children aged 4–15 years in each prefecture each year, including nursery schools, kindergartens, elementary schools, junior high schools, secondary education schools, and special support schools, was obtained from government statistics [nursery schools: statistics of MHLW (30); others: statistics of Ministry of Education, Culture, Sports, Science, and Technology (31)].

### 2.4 Covariates

The total prefectural income per year (32), prefectural mean age (33), and prefectural mean number of decayed, missing, or filled primary teeth (dmft) among 3-year-old children (34) were included as covariates. These variables were selected based on a previous study (14) and theoretical considerations because they reflect socioeconomic conditions, demographic structure, and baseline risk of dental caries at the prefectural level. In addition, in our empirical analyses, this set of covariates was the one for which the conditional parallel trends assumption in the Callaway and Sant'Anna Difference-in-Differences (CSDID) method was satisfied. Data for total prefectural income per year and the prefectural mean dmft of 3-year-old children were obtained for the same year as the outcome. Because the prefectural mean age was collected at 5-year intervals, the nearest preceding survey data were used when data corresponding to the outcome year were not available.

### 2.5 Statistical analysis

Four analytical steps were undertaken. First, we described the trends in outcomes and covariates by survey year. Second, we summarized the timing of dental health ordinance enactment and compared the proportions of children participating in S-FMR programs before and after ordinance enactment within prefectures. Group-level pre- and post-intervention comparisons were performed using the Wilcoxon signed-rank test. Third, we graphed the time trends in the proportion of children participating in S-FMR programs from 2007 to 2018 for each of the three groups.

Fourth, we estimated the impact of prefectural dental health ordinances on the proportion of children participating in S-FMR programs using the CSDID method (35). Difference-in-Differences is a widely used method for evaluating the causal effects of policy interventions. However, traditional two-way fixed-effects regression models have been criticized for producing biased estimates when the timing of interventions varies across units (35). As an alternative, the CSDID method, which offers a more robust statistical framework for settings with staggered treatment adoption, was developed (35). In this study, because the timing of the enactment of dental health ordinances differed among prefectures, the CSDID was considered the most suitable approach. Using the NF group as the control, we estimated the coefficients representing the average treatment effect on the treated (ATT) and 95% confidence intervals (CIs) for the FMR and FA groups.

The parallel trends assumption, requiring that pre-intervention outcome trajectories for the treatment and control groups are similar, was formally assessed using event studies with CSDID. The parallel trends assumption was violated in the preliminary model without covariates. The assumption was satisfied after covariate adjustment, indicating that these variables accounted for the observed differences in the pre-treatment trends. Consequently, analyses were conducted under the conditional parallel trends assumption; that is, after adjusting for covariates, the untreated potential outcomes for the treated and control groups were assumed to follow similar trajectories in the absence of treatment, as permitted by the CSDID framework (35).

Although the interval between the 2015 and 2018 data points was three years, whereas all other intervals were 2 years apart, we coded the 2018 observation as 2017 when constructing the event time for the CSDID analyses to maintain a consistent spacing of periods. All analyses were conducted using Stata SE version 17 (Stata Corp., College Station, TX, USA), with “csdid” package used for CSDID analyses. Statistical significance level was set at *α* = 0.05, and *P*-values of <0.05 were considered statistically significant.

## 3 Results

A total of 39 prefectures were included in the analysis (Figure 1). Of these, 12, 17, and 10 prefectures were classified into the FMR, FA, and NF group, respectively.

Table 1 presents the descriptive statistics of the outcomes and covariates by survey year. At baseline in 2007, the FMR group tended to have a lower prefectural income and a higher mean dmft among 3-year-old children than the FA and NF groups. Across all groups, the proportion of children participating in S-FMR programs increased over time, whereas the mean dmft among 3-year-old children decreased.

**Table 1:** Descriptive statistics for variables by survey year (*n* = 39).

Year	Mean (SD)					
	2007	2009	2011	2013	2015	2018
FMR group (*n* = 12)						
Proportion of children participating in S-FMR programs	0.13 (0.16)	0.16 (0.18)	0.19 (0.21)	0.22 (0.22)	0.29 (0.24)	0.38 (0.27)
Total prefectural income per year (unit: 100 million JPY)	3,882,780 (1,821,213)	3,482,318 (1,628,460)	3,631,218 (1,674,761)	3,699,866 (1,720,123)	3,633,823 (1,642,839)	3,792,982 (1,691,073)
Prefectural mean age (years)	44.65 (1.39)	44.65 (1.39)	46.37 (1.52)	46.37 (1.52)	47.81 (1.63)	47.81 (1.63)
Prefectural mean dmft at 3 years old	1.33 (0.31)	1.15 (0.28)	0.95 (0.26)	0.80 (0.17)	0.80 (0.17)	0.55 (0.13)
FA group (*n* = 17)						
Proportion of children participating in S-FMR programs	0.06 (0.07)	0.07 (0.07)	0.08 (0.08)	0.09 (0.10)	0.11 (0.12)	0.15 (0.15)
Total prefectural income per year (unit: 100 million JPY)	10,000,000 (8,803,411)	9,036,818 (7,894,037)	9,318,401 (8,197,836)	9,715,237 (8,515,491)	9,922,199 (8,886,842)	10,300,000 (9,241,216)
Prefectural mean age (years)	43.70 (1.39)	43.70 (1.39)	45.49 (1.45)	45.49 (1.45)	47.11 (1.44)	47.11 (1.44)
Prefectural mean dmft at 3 years old	1.17 (0.38)	1.03 (0.33)	0.88 (0.29)	0.75 (0.24)	0.66 (0.21)	0.51 (0.16)
NF group (*n* = 10)						
Proportion of children participating in S-FMR programs	0.03 (0.04)	0.04 (0.07)	0.07 (0.12)	0.08 (0.13)	0.09 (0.13)	0.11 (0.14)
Total prefectural income per year (unit: 100 million JPY)	5,464,916 (3,892,543)	4,945,470 (3,613,913)	5,121,643 (3,770,679)	5,264,541 (3,768,055)	5,231,377 (3,763,525)	5,375,635 (3,920,271)
Prefectural mean age (years)	44.48 (1.37)	44.48 (1.37)	46.20 (1.43)	46.20 (1.43)	47.58 (1.44)	47.58 (1.44)
Prefectural mean dmft at 3 years old	1.08 (0.22)	0.94 (0.19)	0.81 (0.15)	0.70 (0.15)	0.65 (0.11)	0.51 (0.08)

S-FMR, school-based fluoride mouth-rinse; FMR group, those explicitly mentioning “fluoride mouth-rinse” in the dental health ordinances; FA group, those explicitly mentioning “fluoride application” in the dental health ordinances; NF group, those without explicit fluoride-related policy language in the dental health ordinances; dmft, the mean numbers of decayed, missing, or filled primary teeth; SD, standard deviation.

1 USD ≈ 150 JPY during the study period.

Table 2 summarizes the year of enactment of prefectural dental health ordinance and the proportion of children participating in S-FMR programs before and after ordinance enactment. The mean year of enactment was similar across the three groups. The difference in outcomes before and after the enactment of the ordinance was the largest in the FMR group (12%), whereas the FA and NF groups showed similar increases (5% each). The Wilcoxon signed-rank test revealed a statistically significant increase in S-FMR participation after ordinance enactment in all three groups.

**Table 2:** Descriptive statistics of dental health ordinance enactment year and the proportion of children participating in school-based fluoride mouth-rinse programs before and after ordinance enactment (*n* = 39).

Classification	Prefecture	Enactment year	Proportion of children participating in S-FMR programs		Difference	*P*-value
			Before enactment	After enactment		
FMR group (*n* = 12)	Mean	2012	0.16	0.28	0.12	<0.001
	Akita	2012	0.25	0.64	0.39	
	Toyama	2013	0.28	0.29	0.01	
	Mie	2012	0.01	0.03	0.02	
	Shiga	2014	0.07	0.11	0.04	
	Kyoto	2013	0.33	0.41	0.07	
	Wakayama	2012	0.10	0.14	0.04	
	Tottori	2013	0.03	0.08	0.05	
	Ehime	2010	0.13	0.20	0.07	
	Saga	2010	0.58	0.75	0.17	
	Nagasaki	2010	0.06	0.31	0.25	
	Kumamoto	2010	0.06	0.30	0.24	
	Oita	2013	0.01	0.12	0.11	
FA group (*n* = 17)	Mean	2012	0.06	0.11	0.05	<0.001
	Aomori	2014	0.04	0.10	0.06	
	Miyagi	2010	0.03	0.04	0.01	
	Yamagata	2013	0.09	0.08	−0.01	
	Fukushima	2012	0.05	0.12	0.07	
	Ibaraki	2010	0.00	0.00	0.00	
	Gunma	2013	0.02	0.02	0.00	
	Saitama	2011	0.01	0.06	0.05	
	Chiba	2010	0.01	0.03	0.02	
	Kanagawa	2011	0.00	0.00	0.00	
	Yamanashi	2008	0.01	0.01	0.00	
	Nagano	2010	0.10	0.13	0.03	
	Gifu	2010	0.12	0.19	0.07	
	Aichi	2012	0.13	0.19	0.06	
	Hyogo	2011	0.02	0.03	0.01	
	Yamaguchi	2011	0.26	0.30	0.04	
	Kagawa	2011	0.15	0.23	0.08	
	Miyazaki	2011	0.07	0.38	0.31	
NF group (*n* = 10)	Mean	2012	0.04	0.09	0.05	<0.01
	Iwate	2013	0.03	0.06	0.03	
	Tochigi	2011	0.06	0.07	0.01	
	Ishikawa	2014	0.01	0.01	0.00	
	Nara	2013	0.02	0.03	0.01	
	Shimane	2010	0.18	0.43	0.25	
	Okayama	2011	0.01	0.02	0.00	
	Hiroshima	2011	0.01	0.01	0.00	
	Kochi	2011	0.02	0.15	0.13	
	Fukuoka	2013	0.00	0.01	0.00	
	Kagoshima	2014	0.04	0.11	0.07	

S-FMR, school-based fluoride mouth-rinse; FMR group, those explicitly mentioning “fluoride mouth-rinse” in the dental health ordinances; FA group, those explicitly mentioning “fluoride application” in the dental health ordinances; NF group, those without explicit fluoride-related policy language in the dental health ordinances.

The *p*-values were calculated using the Wilcoxon signed-rank test.

**Figure 2:**
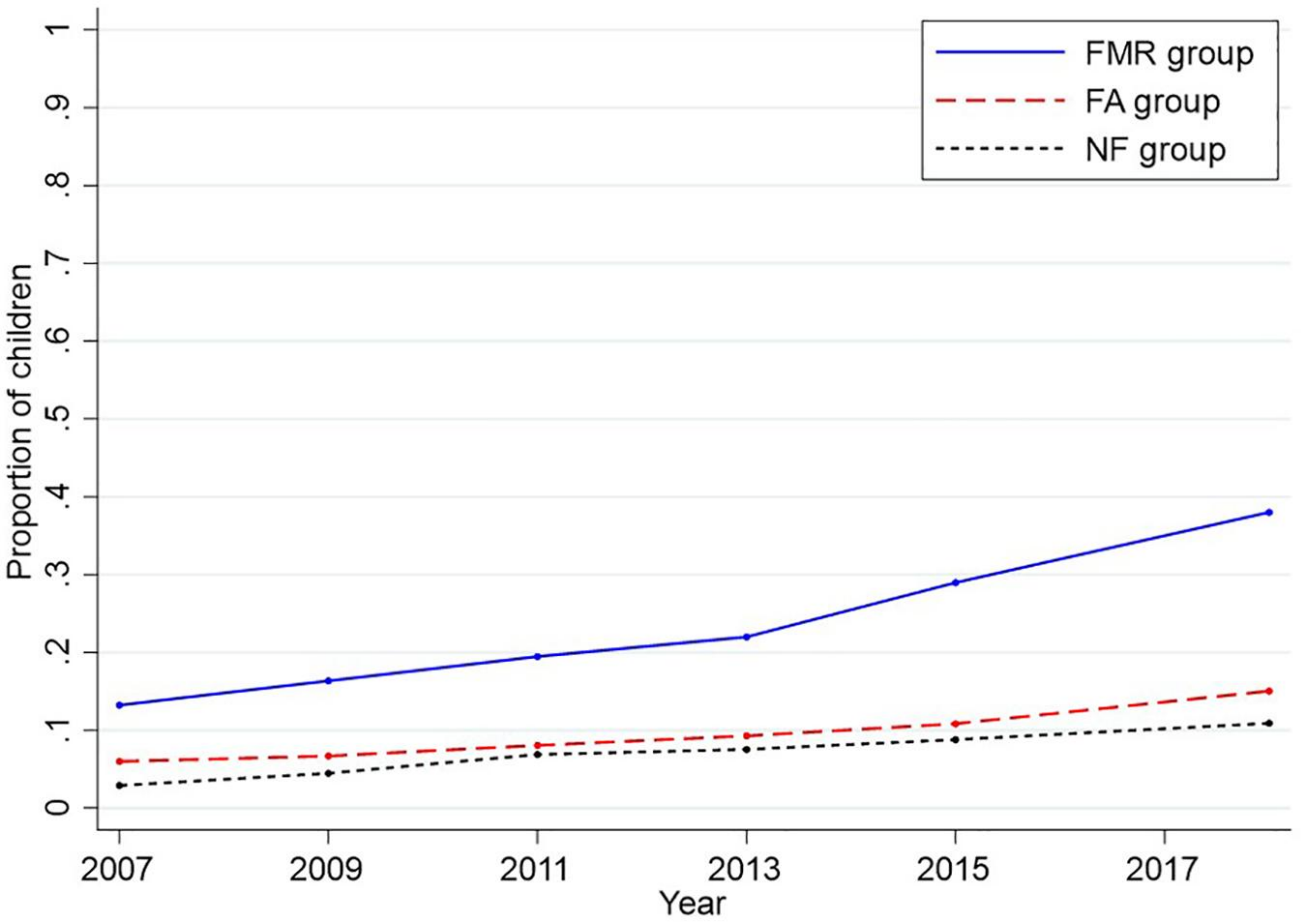
Trends in the proportions of children participating in school-based fluoride mouth-rinse programs from 2007 to 2018 (*n* = 39). FMR group, those explicitly mentioning “fluoride mouth-rinse” in the dental health ordinances; FA group, those explicitly mentioning “fluoride application” in the dental health ordinances; NF group, those without explicit fluoride-related policy language in the dental health ordinances.

Figure 2 shows the trends in the proportion of children participating in S-FMR programs from 2007 to 2018. An increasing trend over time was observed across all groups, with the increase being particularly pronounced in the FMR group, especially in the later years of the study period.

Table 3 presents the results of the CSDID analysis assessing the parallel trends assumption and the ATT of the prefectural dental health ordinance on the proportion of children participating in S-FMR programs. After adjusting for covariates, the pre-trend test yielded non-significant results for both the FMR and FA groups, and the pre-treatment ATTs were close to zero, indicating that the conditional parallel trends assumption was met. Using the NF group as the comparison, the aggregate ATT showed that both the FMR and FA groups experienced statistically significant increases in S-FMR participation. The estimated ATT was 8% (95% CI: 2%–15%) in the FMR group and 5% (95% CI: 0%–9%) in the FA group, with a higher increase observed in the FMR group.

**Table 3:** Results from Callaway and Sant'Anna difference-in-differences estimating parallel trends assumption and the average treatment effect on the treated of the prefectural dental health ordinance on the proportion of children participating in school-based fluoride mouth-rinse programs.

Classification	FMR group (*n* = 12) Control: NF group (*n* = 10)	FA group (*n* = 17) Control: NF group (*n* = 10)
Pre-trend test: *P*-value	0.26	0.13
Aggregate ATT, coefficient (95% CI)	0.08 (0.02–0.15)*	0.05 (0.00–0.09)*
Pre-treatment ATT, coefficient (95% CI)	0.02 (−0.01–0.05)	0.01 (−0.01–0.03)
Post-treatment ATT, coefficient (95% CI)	0.08 (0.03–0.14)**	0.05 (0.01–0.09)*

FMR group, those explicitly mentioning “fluoride mouth-rinse” in the dental health ordinances; FA group, those explicitly mentioning “fluoride application” in the dental health ordinances; NF group, those without explicit fluoride-related policy language in the dental health ordinances; ATT, average treatment effect on the treated; CI, confidence interval.

The total prefectural income per year, prefectural mean age, and prefectural mean number of decayed, missing, or filled primary teeth (dmft) of 3-year-old children were adjusted as covariates.

ATTs represent the change in the proportion of children participating in S-FMR programs.

* *P* < 0.05.

** *P* < 0.01.

**Figure 3:**
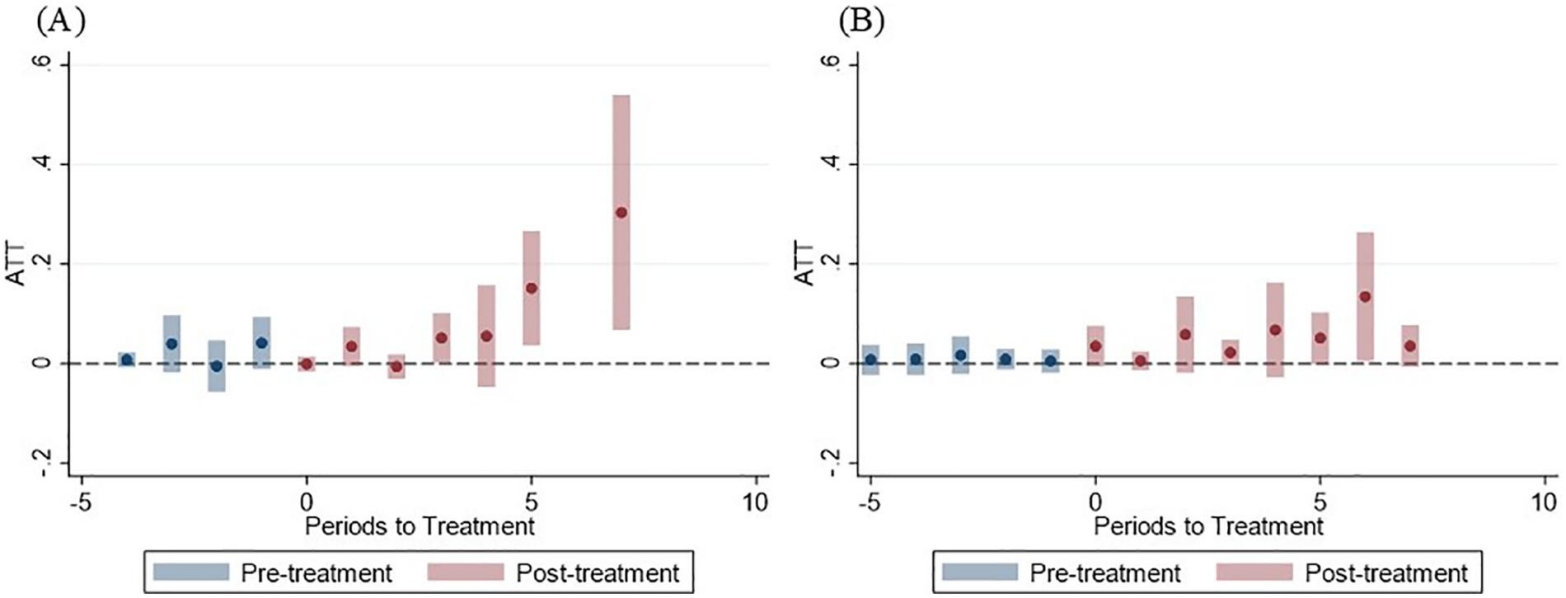
Event study estimates of the dynamic average treatment effect on the treated of the prefectural dental health ordinance on the proportion of children participating in school-based fluoride mouth-rinse programs over years since ordinance enactment. **(A)** FMR group (*n* = 22). **(B)** FA group (*n* = 27). ATT, average treatment effect on the treated; periods to treatment, years since the ordinance was enacted. The total prefectural income per year, prefectural mean age, and prefectural mean number of decayed, missing, or filled primary teeth (dmft) of 3-year-old children were included as covariates. In both **(A,B)**, the NF group served as control. ATTs represent the change in the proportion of children participating in S-FMR programs. The dots show the point estimates and the bars show the corresponding 95% confidence intervals. When the confidence intervals include zero, the effect is not statistically significant at the 5% level.

Figure 3 illustrates the event-study estimates of the dynamic ATT of the prefectural dental health ordinance on the proportion of children participating in S-FMR programs over the years since ordinance enactment. The ATT in the FMR group increased markedly with increasing time since enactment (Figure 3A), whereas in the FA group, the ATT exhibited a more gradual increase over time (Figure 3B).

## 4 Discussion

### 4.1 Summary of key findings

This longitudinal, prefecture-level, quasi-experimental study found that prefectural dental health ordinances including explicit fluoride-related language were associated with a greater increase in the proportion of children participating in S-FMR programs. In particular, ordinances that explicitly specified “fluoride mouth-rinse” showed a larger and progressively strengthening impact on S-FMR participation compared with ordinances that referred more broadly to “fluoride application” or contained no explicit fluoride-related language.

### 4.2 Comparison with previous findings and possible explanations

These findings are partially consistent with implementation research demonstrating that the greater specificity in policy instruments can facilitate effective implementation by reducing ambiguity among local actors (18, 36). Ambiguous policy language allows discretion in interpretation, which may be advantageous during policy formulation but can place an interpretive burden on local implementers and delay the translation of prefectural policy into concrete, on-the-ground implementation (18, 37).

In the present study, ordinances referring broadly to “fluoride application” likely allowed municipalities to interpret the policy in multiple ways, such as emphasizing individual-level measures (e.g., promoting the use of fluoridated toothpaste) rather than implementing resource-intensive, population-based programs like S-FMR. Ordinances without explicit fluoride-related language may have been even more ambiguous. Consequently, these ordinances may have exerted a weaker influence on the dissemination of S-FMR programs.

In contrast, ordinances explicitly specifying “fluoride mouth-rinse” clearly delineates the intended programmatic modality, minimizing the need for local authorities to expend effort on interpretation. This enhanced policy clarity may have enabled local implementers to move directly toward planning and budgeting for the most cost-effective large-scale interventions suitable for school settings, thereby accelerating implementation. Furthermore, explicitly specifying the optimal delivery modality (i.e., S-FMR programs) in the ordinance may have facilitated intersectoral alignment between the health and education departments, thereby streamlining the governance processes required to achieve higher coverage.

Our results differ from those of a previous Japanese cross-sectional study that examined the correlation between the policy language of prefectural dental health ordinances and the proportion of municipalities implementing S-FMR programs in 2011 (38). In that study, no significant association was observed. This lack of association is likely explained by the fact that only 23 prefectures had enacted dental health ordinances at that time and, as suggested by the present results, the effects of such ordinances strengthen over time, making it difficult to detect a significant relationship in an early cross-sectional snapshot.

### 4.3 Implications

These findings have important implications for the implementation of oral health policies. S-FMR programs are supported by strong evidence of their effectiveness, cost-effectiveness, and equity; however, their dissemination remains heterogeneous across regions. Our results suggest that, when prefectural officials draft dental health ordinances, they can strategically use clearer and more explicit fluoride-related wording as an implementation tool to facilitate the uptake of S-FMR programs. Given that only about 13% of children and adolescents participated in S-FMR programs nationally in Japan in 2018 (29), an increase of 8% in the FMR group and 5% in the FA group represents a meaningful public health gain. Thus, for policymakers seeking to expand S-FMR coverage while navigating negotiations with stakeholders, this study provides empirical support for using specific, explicit references to “fluoride mouth-rinse” in ordinance language as a practical lever to promote broader dissemination of the program.

### 4.4 Strengths and limitations of the study

This study has several strengths. First, to our knowledge, this is the first study to use long-term panel data to longitudinally examine the impact of prefectural dental health ordinances on the dissemination of S-FMR programs at the prefectural level. Second, it is the first study to focus explicitly on the policy language of these ordinances and assess their effects over time, thereby providing a more nuanced understanding of how ordinance specificity is associated with implementation. Third, the use of a quasi-experimental CSDID method strengthened the causal interpretation of the effects of different categories of policy language on dental health ordinances.

This study has some limitations. First, as an ecological study based on prefecture-level data, the associations observed at the prefectural level cannot be directly interpreted at the individual-level. Furthermore, the use of aggregate data limited the ability to account for key individual-level determinants of children's oral health, including family and parenting practices and socioeconomic circumstances. However, this design was appropriate given that the objective was to assess the impact of prefectural dental health ordinances at the prefectural level, and because policymakers typically make decisions about ordinance enactment or revision on the basis of aggregated population-level outcomes rather than individual-level effects (20). Second, prefectures without dental health ordinances were excluded because of their small sample size; therefore, the study could not assess the impact of the presence or absence of an ordinance on S-FMR dissemination. Third, one prefecture that changed its ordinance category during the study period was excluded because of the small number of such cases. Therefore, it remains unclear whether revising existing ordinances would produce effects comparable to those observed in this study. Some prefectures revised their ordinance categories only after the end of the study period. Therefore, future research should examine whether revisions to the ordinance language led to subsequent improvements in S-FMR coverage. Fourth, covariates were selected to satisfy the conditional parallel trends assumption. However, unmeasured confounding factors may remain, such as the involvement of dentists in prefectural policymaking and the number of dentists in each prefecture, which could bias the estimated effects. Future studies that comprehensively consider these covariates are warranted. Finally, the generalizability of these findings to other countries and health policies beyond S-FMR programs is limited as this study focuses specifically on Japanese prefectures and S-FMR programs. Specifically, differences in legal systems, governance structures, and oral health care delivery across countries may limit the direct transferability of our findings to other settings. Nevertheless, the findings highlight a potentially generalizable mechanism through which policy design, particularly the use of explicit and actionable language, can facilitate the implementation of preventive health programs. To advance the implementation of evidence-based public health interventions that, like S-FMR programs, are supported by robust evidence yet display substantial regional heterogeneity in uptake, further implementation research explicitly investigating the role of legal frameworks in shaping dissemination and adoption is warranted, and the present study may provide a foundation for such work.

## 5 Conclusion

This quasi-experimental panel study demonstrated that prefectural dental health ordinances explicitly containing fluoride-related policy language were associated with greater dissemination of the proportion of children participating in S-FMR programs in Japan. Ordinances that specifically referenced “fluoride mouth-rinse” showed the largest and progressively strengthening effects, exceeding those of more broadly worded ordinances referring to “fluoride application”. Therefore, incorporating clear and explicit fluoride-related language into municipal dental health may therefore represent an effective strategy for expanding population-based caries prevention programs and reducing regional inequalities in oral health services.

## Data Availability

The raw data supporting the conclusions of this article will be made available by the authors, without undue reservation.
